# Extracellular Vesicles and Cancer Stem Cells in Tumor Progression: New Therapeutic Perspectives

**DOI:** 10.3390/ijms221910572

**Published:** 2021-09-29

**Authors:** Maria Giovanna Scioli, Sonia Terriaca, Elena Fiorelli, Gabriele Storti, Giulia Fabbri, Valerio Cervelli, Augusto Orlandi

**Affiliations:** 1 Department of Biomedicine and Prevention, Anatomic Pathology Institute, University of Rome Tor Vergata, 00133 Roma, Italy; scioli@med.uniroma2.it (M.G.S.); terriacasonia093@gmail.com (S.T.); Elena.Fiorelli@uniroma2.it (E.F.); fabbrigiulia25@gmail.com (G.F.); 2 Plastic and Reconstructive Surgery, Department of Surgical Sciences, University of Rome Tor Vergata, 00133 Roma, Italy; gabriele.storti@uniroma2.it (G.S.); valeriocervelli@virgilio.it (V.C.)

**Keywords:** extracellular vesicles, cancer stem cells, tumor microenvironment, immunosuppression, drug resistance, metastasis, CSC targeting

## Abstract

Tumor burden is a complex microenvironment where different cell populations coexist and have intense cross-talk. Among them, a heterogeneous population of tumor cells with staminal features are grouped under the definition of cancer stem cells (CSCs). CSCs are also considered responsible for tumor progression, drug resistance, and disease relapse. Furthermore, CSCs secrete a wide variety of extracellular vesicles (EVs) with different cargos, including proteins, lipids, ssDNA, dsDNA, mRNA, siRNA, or miRNA. EVs are internalized by other cells, orienting the microenvironment toward a protumorigenic and prometastatic one. Given their importance in tumor growth and metastasis, EVs could be exploited as a new therapeutic target. The inhibition of biogenesis, release, or uptake of EVs could represent an efficacious strategy to impair the cross-talk between CSCs and other cells present in the tumor microenvironment. Moreover, natural or synthetic EVs could represent suitable carriers for drugs or bioactive molecules to target specific cell populations, including CSCs. This review will discuss the role of CSCs and EVs in tumor growth, progression, and metastasis and how they affect drug resistance and disease relapse. Furthermore, we will analyze the potential role of EVs as a target or vehicle of new therapies.

## 1. Introduction

Cancer is a leading cause of death due to its late diagnosis, poor prognosis, drug resistance, and metastasis [1]. As a matter of fact, major research challenges are focused on developing new specific anticancer drugs to overcome tumor recurrence, metastasis, and therapeutic resistance [2]. Several studies have evidenced that the tumor mass is composed of a heterogeneous population of cells, including malignant cancer cells and stromal cells, such as endothelial cells, immune cells, and fibroblasts, as well as a complex network of the extracellular matrix (ECM), which constitutes the tumor microenvironment (TME) [3]. In addition, within the tumor mass, another cell subpopulation with stem features, self-renewal capacity, and the ability to regenerate the tumor cells has been found [4]. That specific cancer cell subpopulation was discovered in 1877 by Virchow’s student, Cohnheim, demonstrating that when those cells are injected into a xenograft mouse model, they form a new tumor mass [5]. Initially, that cancer cell subpopulation has been named as tumor-initiating cells (TICs). It is not yet clear if those cells derive from a stem cell that underwent malignant transformation or if they are the result of stem-program activation and dedifferentiation of a tumor cell [6,7,8]. However, due to those intrinsic properties, nowadays they are often called cancer stem cells (CSCs) [9]. In the last few years, tumorsphere formation in vitro and limiting-dilution tumorigenicity assays in vivo represent the golden standards for creating CSC models [10]. Many studies have demonstrated that CSCs can also be isolated by a cluster of differentiation (CD) markers, such as CD133, CD44, CD24, CD90, and CD34 [6]. Later, the enzyme aldehyde dehydrogenase (ALDH or ALDH-1) and epithelial cell adhesion molecule (EpCAM) were identified as other CSC markers [6,11,12]. It has been also reported that the expression of CSC markers can vary according to the tumor type [13]. Furthermore, CSCs show a dysregulation in pathways related to stemness and self-renewal, such as wingless/integrated (Wnt), phosphoinositide 3-chinasi/protein kinase B/forkhead transcription factors of the O class (PI3K/Akt/FOXO), transforming growth factor-beta (TGF-β), and Notch [14,15,16,17]. These dysregulated pathways give CSCs the ability to induce epithelial–mesenchymal transition (EMT), self-renovation, evasion from the immune system, metastasis, and resistance to conventional therapies [18,19]. CSCs are capable of acquiring both an epithelial/proliferating and a mesenchymal/invasive phenotype, demonstrating a great plasticity to switch between these two phenotypes, and thus probably playing a crucial role in EMT [20,21]. CSCs have the ability to evade the immune system, and it is due to the secretion of a variety of cytokines and extracellular matrix components that promote M2 polarization of resident macrophages and inhibit T cell response [22,23,24].

Current chemotherapies and radiotherapies are able to destroy the tumor bulk but not CSCs, leading to cancer recurrence [19]. Drug resistance may also be due to specific CSC features, such as the upregulation of drug efflux pumps, increased ability of DNA damage repair, reactive oxygen species (ROS) elimination, and their dormancy state [25,26,27]. TGF-β signaling seems to be responsible for the quiescent state of CSCs in different breast cancer types [28]. Furthermore, during the antiproliferative treatment, CSCs can escape from dormancy and reactivate themselves to repopulate the tumor bulk [29].

CSCs are able to interact with the surrounding stromal cells and non-CSC tumor cells by the paracrine secretion of several molecules, including growth factors, cytokines, inflammatory mediators, and nucleic acids, which can be released through extracellular vesicles (EVs) [30,31]. EVs are nanosized particles that can be divided into three main types: exosomes, microvesicles, and apoptotic bodies [32]. Exosomes are a homogeneous population with a size between 50 and 100 nm that originate from the endosomal system and have a cuplike shape [32]. Microvesicles (MV) have a 100–1000 nm size and derive from outward blebbing of the plasma membrane [32]. Apoptotic bodies are released during the last step of apoptosis and have a size between 400 and 1000 nm [32]. Different methods have been described for EV isolation, such as ultracentrifugation, ultrafiltration, precipitation, and immunoselection by magnetic beads, ELISA, or flow cytometry for EV-specific markers CD9, CD63, and CD81 [33,34].

The generation of exosomes initiated from early endosomes, which were formed by invagination of the plasma membrane [35]. After invagination, their content can be recycled to the plasma membrane or incorporated into intraluminal vesicles (ILVs) and then transformed into multivesicular bodies (MVBs) [36]. During this process, cytosolic proteins, nucleic acids, and lipids are packed into these vesicles [36]. The specific sorting process of a protein cargo into exosomes is regulated by various proteins, such as endosomal sorting complexes required for transport (ESCRT), which recognizes ubiquitinated proteins and facilitates their inclusion into ILVs of MVBs [37,38]. Once formed, MVBs fuse with lysosomes that can degrade MVB content or fuse with the plasma membrane to form exosomes [39]. Once exosomes are released into the extracellular space, they can be uptaken by target cells (recipient cells) through endocytosis [40].

Regarding MVs, their biogenesis is still not very clear and seems to involve several mechanisms [41]. It has been reported that these vesicles are formed by the outward blebbing and fission of the plasma membrane, and this process could be regulated by a combination of factors, such as the redistribution of phospholipids and contraction of the actin–myosin machinery [42]. Moreover, the release of MVs can be induced by external factors, such as the influx of calcium that stimulates the redistribution of phospholipids and consequently increases the release of MVs. It was been reported that hypoxia can promote MV release through hypoxia-inducible factor (HIF)-dependent expression of Ras-related protein Rab-22A (RAB22A) [43,44].

By transferring proteins, lipids, and nucleic acids into target cells, EVs can orchestrate cell–cell communication, especially in the tumor bulk, where CSCs can exert their intrinsic properties, such as self-renewal, chemoresistance, and escape from immune response [45,46]. For instance, CSCs release EVs that can be uptaken by stromal cells, non-CSC tumor cells, immune cells, and endothelial cells, inducing stemness regain, anticancer drug resistance, metastasis, angiogenesis, and immunosuppression [47,48]. In particular, fibroblasts can be transformed, through the uptake of CSC-EVs, into cancer-associated fibroblasts (CAFs), promoting tumor progression and metastasis [49]. At the same time, stromal cells and non-CSC tumor cells can also secrete EVs, favoring cell interplay and procancer functions in the tumor bulk [50]. Therefore, there currently is a great interest in the development of new anticancer therapies based on EV-targeted strategies.

In this review, we will discuss in detail the underlined mechanisms involved in the protumoral activities of EVs, especially the ones derived from CSCs, and the recent advances of new EV-targeted anticancer therapies.

## 2. Protumoral Effects of CSC-Derived EVs on Non-CSC Tumor Cells

### 2.1. Stemness and Metastatic Phenotype

Emerging evidence revealed the presence of a cross-talk between CSCs and non-CSC tumor cells, which play a critical role in tumor progression [47]. Several studies have proven the key role of EV cargos released by CSCs in the tumorigenicity, metastasis, and stemness regain of non-CSC tumor cells [51,52]. Many works have shown that CSCs, due to their dysregulated pathways, are capable of giving rise to secondary tumors [53,54]. Metastasis is strongly associated with ECM remodeling and EMT, in which cancer cells acquire features of mesenchymal cells and express markers such as N-cadherin, vimentin, matrix metalloproteinases (MMPs), such as MMP-9, MMP-1, and MMP-2 [55,56]. EVs are leading actors in primary cancer metastasis, premetastasis niche formation, and colonization of cancer cells at metastatic sites [50]. Recently, several studies have investigated the effects of CSC-derived EVs, through the release of proteins, microRNAs (miRNAs), and long noncoding RNAs (lncRNAs), in the metastatic transformation of non-CSC tumor cells [57]. In this regard, it was reported that miR-210-3p, contained in tumor exosomes, plays an important role in the metastatic process of prostatic, renal, and lung carcinoma [58,59,60]. Based on those findings, Wang et al. investigated the role of lung CSC-derived exosomes in the regulation of a prometastatic phenotype in lung cancer cells (non-CSCs) [61]. They purified exosomes from CSCs derived from adenocarcinomic human alveolar epithelial cells (A549) and lung squamous cell carcinoma (NCI-H1703). The treatment of lung cancer cells with CSC exosomes promoted their migration and invasion. In addition, the coincubation of lung cancer cells with exosomes derived from miR-210-3p-transfected CSCs increased metastatic abilities. They identified fibroblast growth factor receptor-like 1 (FGFRL1) as a possible target for miR-210-3p. Indeed, FGFRL1 silencing in lung cancer cells increased their migratory and invasive potential, whereas its overexpression in lung cancer cells decreased those abilities [61]. This study demonstrated that CSCs, through the release of exosomes rich with miR-210-3p, induce a prometastatic phenotype in lung cancer cells, downregulating FGFRL1, which may act as a metastatic inhibitor, suggesting new possible therapeutic targets in lung cancer.

CSCs have also been reported to exert a crucial role in the prometastatic induction of clear cell renal cell carcinoma (CCRCC), in which over 30% of patients suffer from metastasis, especially in the lungs [62,63]. In this regard, Wang et al. investigated the effects of CSC exosomes in the induction of EMT and lung metastasis on renal carcinoma cell lines as well as the role of miR-19b-3p, already proven to be involved in CCRCC-derived lung metastasis [64,65].

They collected CCRCC cells from tissue samples (from nonmetastatic and lung metastatic CCRCC patients) in order to isolate CSCs. Then, CSC-derived exosomes were purified and coincubated with renal adenocarcinoma cell line (ACHN) and 786-O. The latter showed increased viability, migration, and invasion, as well as higher levels of EMT markers, such as N-cadherin, vimentin, and Twist, especially with exosomes isolated from metastatic patients. Moreover, they observed that CSC exosomes were rich with miR-19b-3p and ACHN and 786-O cells treated with those exosomes. They identified phosphatase and tensin homolog (PTEN) as a putative target of miR-19b-3p, and CCRCC cell lines transfected with miR-19b-3p displayed a reduced PTEN expression indeed. They also set up an in vivo experiment in which nude mice were coinjected with CSC exosomes and ACHN or 786-O cells, demonstrating that CSC exosomes promoted tumor growth and lung metastasis. To confirm that metastatic CSC exosomes have a greater cancerogenic potential than those from nonmetastatic CSCs, they analyzed the cell fusion capacity and tissue/organ targeting of exosomes under both in vitro and in vivo conditions. They demonstrated that metastatic CSC exosomes show a greater efficiency to fuse with ACHN and 786-O cells and are more densely aggregated in the lungs, compared with nonmetastatic CSC exosomes. Then, they injected metastatic CSC exosomes in CCRCC-bearing mice, in which the integrin CD103, involved in organotropic metastasis [66], was previously inhibited. They demonstrated that metastatic CSC exosomes were no longer capable of reaching the tumor site and lungs. They also found that blood samples from metastatic CCRCC patients showed higher levels of CD103^+^ exosomes. This study revealed the key role of CSC exosomes in the promotion of EMT and metastasis through the release of miR-19b-3p, as well as the crucial function of CD103 in the metastatic process, suggesting these two markers as targets in metastatic CCRCC patients.

CSCs also contribute to the development of glioma, an aggressive intracranial tumor, through the activity of many factors contained in their exosomes, lncRNAs among them [67,68]. Based on previous data, Zhang et al. analyzed the role of CSC and non-CSC exosomes in the tumor progression of two glioma cell lines, pointing to the critical role of a particular exosomal lncRNA, named small nucleolar RNA host gene 16 (SNHG16) and already known to promote tumor development [69,70,71]. They collected surgical specimens of glioma from patients showing that SNHG16 was upregulated. From these specimens, they isolated CSCs and non-CSC tumor cells as well as their exosomes, demonstrating that SNHG16 was higher expressed in CSC exosomes. To clarify the role of this lncRNA in glioma development, they set up an in vitro experiment in which two glioma cell lines, such as SHG44 and U251, were incubated with exosomes isolated from SNHG16-overexpressing CSCs or SNHG16-knockdown CSCs. They found that SNHG16 is internalized by non-CSC tumor cells, in which the SNHG16 overexpression increased their viability, migration, and invasion. Subsequently, the authors analyzed the correlation between SNHG16 and toll-like receptor 7 (TLR7), which are activated by single-stranded oligonucleotides and involved in various disease progressions [72]. TLR7 activates nuclear factor kappa B (NFκB) and c-Myc through myeloid differentiation factor 88 (MyD88) in different cancer cells [73,74]. Zhang et al. showed that when SNHG16 is overexpressed, TLR7 is downregulated (si-TLR7) and pulled down in the strand of SNHG16. In addition, they demonstrated that SNHG16 overexpression promoted NFκB activity and the increase in p65, p50, and c-Myc, whose expression is reduced by SNHG16 knockdown. The coinjection of SHG44 and U251 cells with exosomes from SNHG16-overexpressing CSCs into nude mice promoted glioma progression, whereas the inoculation with SHG44 and U251 transfected with si-TLR7 or si-MyD88 reverted those effects by decreasing NFκB activity and c-Myc expression. This study demonstrated that CSC exosomes, releasing SNHG16, promoted glioma progression by activating the TLR7/MyD88/NFκB/c-Myc signaling pathway, suggesting those markers as possible targets for glioma treatments. However, it is important to highlight that SNHG1 is not the only factor secreted by glioma CSCs, and therefore, more studies are required to identify all the potential agents involved in glioma progression in order to target appropriate molecules.

Another study by Sun et al. identified another important factor involved in glioma progression [75].

They explored the ability of glioma stem cell (GSC) exosomes to reprogram non-GSC glioma cell lines through the release of Notch-1, known to be important in the maintenance of the cancer-stem-like phenotype and upregulated in glioma stem cells (GSCs) [76]. They conducted an in vitro study using human glioblastoma multiforme (GBM) cell line (WJ1) from which they isolated GSCs and subsequently their exosomes. Glioma cell lines U251 and U87 (non-GSCs) incubated with GSC exosomes showed an increase in proliferative activity, neurosphere formation (stemness feature), cell invasion, and tumorigenicity. Notch-1 expression was upregulated in GSC exosomes compared with exosomes derived from U251 and U87 cells. Moreover, GSC exosomes increased Notch-1, as well as stemness-related proteins such as CD133, nestin, octamer-binding transcription factor 4 (Oct4), and SRY-box transcription factor 2 (Sox2), in treated non-CSC glioma cells. When U251 and U87 cells are treated with si-Notch-1-transfected GSCs-derived exosomes, the expression of those proteins decreased. Therefore, glioma cells retain stemness and increase tumorigenicity through the transfer of Notch-1 in GSC exosomes.

CSCs have also been reported to be involved in the metastasis and stemness regain of colorectal cancer (CRC) that often shows recurrence and metastasis after surgery [77]. Circular RNAs (circRNAs) are noncoding RNAs without 5′ and 3′ ends that were proven involved in CRC progression by activating different pathways, such as Wnt/β-catenin [78]. In this regard, Zhao et al. explored the role of a new circRNA, named circ-ABCC1, which they expected to find as CSC exosome cargo, coincubating CSC exosomes with malignant CRC cell lines [79]. In particular, human colorectal adenocarcinoma cells (Caco2 and HCT15) were used to isolate CD133^+^ cells (CSCs) and subsequently their exosomes. When CRC cell lines were incubated with CSC exosomes, they showed a higher ability of tumorsphere formation and migration, as displayed by circ-ABCC1-transfected Caco2 and HCT15. To assess the involvement of the Wnt pathway by circ-ABCC1 in tumorsphere formation, Zhao et al. added KYA1797K (a Wnt inhibitor) to circ-ABCC1-transfected Caco2 and HCT15 that were no longer able to form tumorspheres. The entry of β-catenin into the nucleus can activate the Wnt pathway [80]. As a matter of fact, the overexpression of circ-ABCC1 in Caco2 and HCT15 led to an increased nuclear expression of β-catenin that bound to circ-ABCC1 [79]. This study suggests that CSC exosomes favor stemness regain and metastasis progression in CRC cells through the release of circ-ABCC1, which in turn activates the Wnt pathway. This conclusion points to these factors as potential new CRC targets.

### 2.2. Drug Resistance

As previously reported, another important feature of CSCs is their resistance to standard chemotherapy and radiotherapy [81]. Chemotherapy is effective in destroying non-CSC tumor cells but not CSCs that remain quiescent at the tumor site, surviving and expanding themselves [82]. Several mechanisms have been assumed to be responsible for CSC-mediated drug resistance, such as EMT, drug transport proteins, dormancy, and DNA repair. Regarding EMT, some pieces of evidence support a correlation between EMT activation and drug resistance [83]. For example, gefitinib, a tyrosine kinase inhibitor, was able to stimulate lung cancer cells to acquire a mesenchymal phenotype through Notch-1 signaling activation [84]. Moreover, mesenchymal phenotype acquisition makes cells less susceptible to an epidermal growth factor receptor (EGFR) inhibitor [84]. Besides EMT, the high expression of stem cell markers (i.e., Oct4, Nanog, Sox2, Lgr5) and drug transporter proteins (i.e., MDR1, ABCG2, ABCB1) can contribute to CSC therapy resistance [85,86]. It has also been demonstrated that the activity of ALDH, a cytosolic enzyme that protects cells against the cytotoxic effects of ROS, is highly expressed in leukemic CSCs and plays a key role in chemotherapy resistance [87]. Furthermore, the quiescent state of CSCs can represent an additional protection to anticancer therapies [88].

Many studies suggest that EVs derived from CSCs contain active molecules that, when internalized by non-CSC tumor cells, can activate drug-resistance-related signaling pathways [89]. For instance, in patients suffering from pancreatic cancer, a prolonged treatment with gemcitabine (GEM) induced the development of chemoresistance [90]. In this regard, Yang et al. hypothesized that exosomes released by CSCs can confer drug resistance to pancreatic malignant non-CSC tumor cells, in particular through the uptake of miR-210, a drug-resistance-related miRNA [91].

In particular, they used human pancreatic cancer-derived cell lines (BxPC-3) GEM resistant and GEM sensitive (BxR and BxS, respectively) to isolate CSCs. CSCs were treated with GEM, demonstrating that BxR-CSCs were more resistant than BxS-CSCs because they expressed higher levels of self-renewal and drug-resistance markers. Then, they isolated exosomes from BxR-CSCs and BxS-CSCs and incubated them with the human pancreatic cancer cell line PANC-1 (sensitive to GEM). Exosomes from BxR-CSCs increased the expression of drug resistance-related proteins, such as multidrug resistance protein 1 (MDR1), Y-box binding protein 1(YB-1), and breast cancer resistance protein (BCRP), and the proliferation, migration, and tube formation of PANC-1 cells. Moreover, they observed that the treatment with GEM increased miR-210 expression in a dose-dependent fashion in BxS and PANC-1 cells cocultured with BxR-CSC-derived exosomes. They also proved that BxR-CSC exosome treatment increased the expression of miR-210 in PANC-1 cells. To determine how miR-210, transferred by BxR-CSC exosomes, conferred drug resistance and stimulated tumor progression, they transfected PANC-1 with a miR-210 inhibitor or mimic, exposing cells to GEM at increasing concentrations. Results showed that the inhibition of miR-210 induced a cell cycle arrest at the G2/M phase, whereas miR-210 overexpression inhibited GEM-induced apoptosis and increased the phosphorylation of ribosomal protein S6 kinase beta-1 (S6K1), a downstream target of the mechanistic target of rapamycin (mTOR). The same results were obtained in vivo, as they observed a greater tumor growth in nude mice injected with BxS overexpressing miR-210. This study suggests that CSC exosomes play a pivotal role when it comes to the drug resistance of pancreatic cancer, particularly because they transfer miR-210, a new potential therapeutic target.

It has been hypothesized that drug resistance in breast cancer could be partially referred to CSC exosomes and their content uptake [92]. Among miRNAs, miR-155 seems to be associated with breast cancer drug resistance [93]. It has been reported that miR-155 binds FOXO-3a 3′-UTR, regulating drug response, and is also implicated in the loss of CCAAT/enhancer-binding protein beta (C/EBP-β) activity and TGF-β-induced EMT, invasion, and metastasis in breast cancer cells [94,95]. Based on those findings, Santos et al. investigated the correlation between the EMT-mediated chemoresistance and the transfer of miR-155 by CSC exosomes in malignant breast cancer cell lines [96]. First, the human breast cancer cell lines MCF-7 and MDA-MB-231 were treated with doxorubicin (DOX) and paclitaxel (PTX) to confirm their chemosensitivity. Then, they obtained DOX-resistant and PTX-resistant variants with a mesenchymal phenotype by a prolonged culture at increasing drug concentrations. In addition, they generated CSCs in vitro by mammosphere formation. Both CSCs and chemoresistant cells expressed CSC markers and an increased miR-155 in their exosomes. When breast cancer chemosensitive cells were incubated with exosomes from CSCs and chemoresistant cells, they showed an increase in migration, miR-155 expression, EMT markers, and resistance to DOX and PTX. To understand whether the chemoresistance is attributed to exosome-mediated miR-155 transfer, exosomes from miR-155 mimic-transfected MCF-7 were incubated with nontransfected MCF-7 cells, which in turn showed increased levels of miR-155. Furthermore, they observed a reduction of TGF-β, C/EBP-β, and FOXO3a in miR-155-transfected MCF-7, which became resistant to DOX and PTX [96]. The critical role of CSCs and their transmission of exosome-mediated resistance suggests new potential therapeutic strategies against chemoresistance in breast cancer patients.

CSCs are also resistant to radiotherapies and their induced damage [97]. There are different signaling pathways involved in radioresistance, such as phosphatidylinositol3-kinase (PI3K), mitogen-activated protein kinase (MAPK), sirtuin (SIRT) pathways, Wnt/β-catenin signaling, interleukin 22 receptor subunit alpha 1/signal transducer and activator of transcription 3 (IL22RA1/STAT3) signaling, and sonic hedgehog signaling [98]. In regard to breast cancer, radioresistant MDA-MB-231 breast cancer cells (RT-R-MDA-MB-231 cells) are malignant cells with a high metastatic activity, proliferation rate, and expression of CSC and EMT markers [99]. Paramanantham et al. hypothesized the presence of an altered signal in those cells and set up an antibody array analysis on both radioresistant MDA-MB-231 and parental ones (p-MDA-MB-231) [100]. They found an upregulation of MAPK1, dual specificity protein kinase (CLK1), and fibroblast growth factor 22 (FGF22) and a downregulation of caspase-3 in RT-R-MDA-MB-231 cells. Gene ontology enrichment analysis revealed that MAPK1 was the main signaling pathway involved in the radioresistance of RT-R-MDA-MB-231 cells. To better understand its involvement in radioresistance, they treated RT-R-MDA-MB-231 and p-MDA-MB-231 cells with a mitogen-activated protein kinase/extracellular signal-regulated kinase (MEK/ERK) inhibitor, PD98059. RT-R-MDA-MB-231 cells displayed a reduced survival and an increased death compared with p-MDA-MB-231 cells, suggesting that ERK signaling could be important in the survival of RT-R-MDA-MB-231 cells. Moreover, they found that RT-R-MDA-MB-231 cells showed mitochondrial fission and swelling of nuclei, whereas p-MDA-MB-231 cells showed shrinkage and fragmentation of nuclei, suggesting that ERK inhibition induces cell death in both cells but with different mechanisms. Molecular analysis revealed that ERK inhibition induced the cleavage of poly(ADP-ribose) polymerase 1 (PARP-1) and caspase-3 (apoptosis markers) in p-MDA-MB-231 cells and the activation of the apoptosis-inducing factor (AIF), which positively regulates cyclophilin A (CypA) protein (biomarker of necroptosis), in RT-R-MDA-MB-231 cells. In the latter, ERK inhibition markedly suppressed CSC and EMT markers [100]. Therefore, ERK signaling is strongly involved in the radioresistance of MDA-MB-231 breast cancer cells, suggesting that its targeting may offer a potential therapeutic strategy.

## 3. Cross-Talk between CSCs and the Tumor Microenvironment via EV Transfer

Particular attention in tumor progression must also be addressed to TME, which is mainly composed of stromal cells, such as fibroblasts and mesenchymal stem cells (MSCs), vascular cells, immune cells, and ECM [101], which altogether orchestrate cancer homeostasis. CAFs are a major component of the tumor stromal compartment. CAFs are a heterogeneous population of fibroblasts that transforms into a cancer-associated fibroblastic population, under tumor stimuli. CAFs show a highly proliferative capacity, a contractile phenotype, and an increased secretion of ECM, proteases, and growth factors, which promote tumorigenesis and metastasis [102,103,104].

TME plays a critical role in the maintenance of CSC stemness and self-renewal; on the other hand, CSCs help to create this favorable microenvironment [105]. It has also been suggested how this cross-talk can occur via EV transfer and the regulation of important processes, such as angiogenesis, immunotolerance, proliferation, and clonogenicity [105]. There is a tight connection between cancer and the environment, as well as cell–cell interaction and soluble factor release. Proteins, growth factors, and genetic materials, such as miRNAs, can be exchanged via exosome release and uptake [106]. They are able to stimulate tumor progression through chemoresistance, metastasis formation, neoangiogenesis, and immunosuppression [107].

### 3.1. EV-Mediated Communication between CSCs and Stromal Cells

An increasing amount of evidence suggests that CSC-derived exosomes can modulate the stromal compartment, favoring tumor progression and metastasis formation [108]. In a recent study by Zhang et al. [49], the authors analyzed the effects of exosomes derived from fibroblast-derived CSCs on the proliferation, migration, and invasion of human parental fibroblasts (FBs). In particular, the treatment with CSC-derived exosomes induced FB proliferation, migration, and invasive capabilities, increasing the expression of metalloproteinase MMP2 and MMP9, smooth muscle alpha-actin (α-SMA), vimentin, and fibroblast activation protein (FAP). The expression of these proteins demonstrated that FBs, under exosome transfer, underwent transformation into CAFs. Similarly, Lindoso et al. [109] studied the effects of CSC-derived EVs, isolated from renal carcinoma, on bone marrow mesenchymal stromal cells (MSCs), which had already been reported to be protumorigenic in osteosarcoma cells [110]. MSCs, under stimulation with CSC-derived EVs, showed an increased expression of MMP1 and MMP3, genes associated with cell migration such as CXC chemokine receptor type (CXCR4, CXCR7); matrix remodeling such as collagen, type IV, alpha 3 (COL4A3); angiogenesis; and tumor growth such as interleukin 8 (IL-8), osteopontin, and myeloperoxidase. Moreover, EV-treated MSCs stimulated tumor cell migration and vessel-like formation. The coinjection of EV-treated MSCs with renal cancer cells in mice with severe combined immunodeficiency (SCID mice) proved to favor tumor development and vascularization, confirming the pivotal role of CSC-derived EVs in the regulation of the stromal microenvironment and tumor growth.

On the other hand, Sansone et al. [111] found that in a breast cancer xenograft model, CAFs can deliver, by exosome transfer, mitochondrial DNA to CSCs. CAF-derived EVs induced a metabolic reactivation of CSCs, which showed a dormancy state and an impaired oxidative phosphorylation under hormonal therapy. Moreover, a study by Figueroa et al. [112] showed the effects of exosomes derived from glioma-associated human mesenchymal stem cells (GA-hMSCs), a stromal component of glioblastoma, on glioma-stem-like cells (GSCs). They showed that GA-hMSC exosomes stimulated the proliferation and clonogenicity of GSCs in orthotopic xenografts, demonstrating their protumorigenic activity. In particular, they demonstrated that the oncogenic effects were due to the presence of miR-1587 contained in the exosomes that decreased the expression of tumor suppressor nuclear receptor corepressor 1 (NCOR1) in GSCs. These findings support the bidirectional communication between CSCs and the stromal microenvironment via EV transfer and its fundamental role in tumor development and progression.

### 3.2. The Immunosuppressive Role of CSC-Derived EVs

Eluding the immune system is one of the main capabilities of cancer cells, and it is fundamental for tumor progression [113]. For instance, an increase in the number of immunosuppressive cells, such as CD4+/CD25+/FoxP3+ regulatory T cells and myeloid-derived suppressor cells, has been reported in glioma tumors [114,115]. Moreover, GSCs stimulated immunosuppressive macrophages/microglia [116]. In this context, Yang et al. [117] demonstrated that the treatment with GSC-derived EVs modulated the inflammatory response of lipopolysaccharide (LPS)-stimulated microglia cells, inducing them to secrete IL-6, IL-8, tumor necrosis factor (TNF)-α, and cytokines, known to be involved in glioma growth, angiogenesis, and resistance to chemotherapy, radiation, and apoptosis [118]. They found that this response in microglia cells was due to the uptake of EV-derived lncRNA lung adenocarcinoma transcript 1 (MALAT1), which inhibited miR-129-5p and upregulated high mobility group box protein 1 (HMGB1), protecting microglia from injury and inflammation. Domenis et al. investigated the immunosuppressive effects of GSC-derived exosomes on isolated peripheral blood mononuclear cells (PBMCs) from healthy patients. In particular, those authors found that exosome-treated PBMCs prevented the activation and proliferation of T cells [119]. Furthermore, Mirzaei et al. investigated the interactions between T cells and the highly radio- and chemoresistant stemlike brain tumor initiating cells (BTICs) [120]. Notably, conditioned media from activated T lymphocytes inhibited BTIC growth, but when in cocultures, BTICs released through exosomes extracellular matrix protein tenascin-C (TNC), which prevented T cell activation and proliferation through the binding with α5β1 and αvβ6 integrins. TNC depletion in exosomes partially restored T cell activity. In addition, circulating exosomes from glioblastoma patients showed higher levels of TNC and T cell-suppressive activity, compared with those from healthy subjects.

In colon cancer, Hwang et al. [121] studied cell–cell interactions between immune cells and colorectal cancer stem cells (CRCSCs) obtained from spheroids of mouse colon cancer CT26 cells. In particular, CRCSC-bearing mice showed an increased number of immunosuppressive CD11b+/Ly6Ghigh/Ly6Clow neutrophils that localized in the bone marrow together with CRCSC exosomes. They found that the treatment with CRCSC exosomes, containing specific miRNAs, favored bone marrow-derived neutrophil survival by increasing the expression of interleukin-1β (IL-1β). In addition, the coinjection of exosome-primed neutrophils with parental colon cancer CT26 cells increased in vivo tumorigenesis and metastatic spreading counteracting IL-1β inhibition.

The pivotal role of macrophages in malignant tumor development and progression has become apparent [122]. The polarization and differentiation of macrophages into the tumor-suppressing M1 and protumoral M2 macrophages is a crucial phenomenon in the tumor microenvironment, in particular for immunosuppression [123]. Gabrusiewicz et al. [124] found that GSC-derived exosomes (GDEs) showed a higher affinity for monocytes, (i.e., macrophage precursors). In addition, GDEs were internalized in the monocyte cytoplasm, determining a cytoskeleton reorganization of the actin and the switch into M2 phenotype. The analysis of the GDE content revealed the presence of members of the signal transducer and activator of the transcription 3 (STAT3) pathway responsible for the M2 polarization. Moreover, monocytes treated with GSC exosomes showed an increased expression of programmed death-ligand 1 (PD-L1), which can inhibit the immune response by activating the receptor of programmed cell death protein 1 (PD-1) [125].

### 3.3. The Proangiogenic Properties of CSC-Derived EVs

EVs can have a key role in the tumor microenvironment neoangiogenesis [126]. In a study by Grange et al. [127], the author investigated the effects of renal-CSC-derived exosomes on vessel formation. Human umbilical vein endothelial cells (HUVECs) treated with CSC-derived MVs were able to form more tubular structures compared with those treated with MVs from non-CSC tumor cells. In immunodeficient mice, the injection of CSC-derived MVs significantly favored angiogenesis and metastatic spreading in lungs. Those findings suggest the influence of CSC-derived MVs in vascularization and tumor progression. Interestingly, Vera et al. [128] studied the effects of EVs from cisplatin-treated ovarian cancer spheroid (OCS) on bone-marrow-derived mesenchymal stem cells (BM-MSCs). Notably, BM-MSCs incubated with cisplatin-treated OCS-EVs expressed higher levels of IL-6, IL-8, vascular endothelial growth factor A (VEGFA), MMP1, 2, and 3, as well as increased migration. Moreover, conditioned medium from BM-MSCs incubated with cisplatin-treated OCS-EVs significantly increased the angiogenic properties of HUVECs compared with those without cisplatin treatment. A study by Conigliaro et al. concerning hepatocarcinoma [129] also revealed that exosomes from liver CSCs stimulated the angiogenic phenotype and cell-to-cell adhesion in HUVECs through the transfer of long intergenic non-protein-coding RNA 8 (lncRNA H19), thus demonstrating the key role of lncRNA H19 in tumor angiogenesis and suggesting it as a putative therapeutic target in hepatocellular carcinoma.

Finally, Wang et al. [130] found that in glioma GSC-derived EVs induced proliferation, migration, tube formation, and angiogenesis of human brain microvascular endothelial cells by releasing miR-26a, which upregulates the PI3K/Akt signaling pathway and downregulates PTEN. The chicken chorioallantoic membrane (CAM) assay also confirmed these proangiogenic effects of miR-26a.

Altogether, those results highlight the crucial role of EV transfer in the tumor–stroma communication, regulating cancer development and progression. The inhibition of this cross-talk represents a promising strategy for new anticancer therapies to overcome issues such as drug resistance and immunosuppression. However, a deeper knowledge about the specific EV content and the biomolecular mechanisms activated is still required.

## 4. Cross-Talk between Non-CSC Tumor Cells and the Tumor Microenvironment via EV Transfer

EVs also play a critical role in the communication between non-CSC tumor cells and stromal cells [50]. In particular, non-CSC-tumor-derived EVs exert their effects on neighboring cells, such as endothelial cells, immune cells, and fibroblasts, contributing to vascularization, tumor growth, local invasion, and suppression of the immune system [50]. Several studies have investigated the functions of non-CSC-tumor-derived EVs and their miRNA content in cancer angiogenesis, growth, and metastatic process [131,132]. Lin et al. analyzed the expression of 19 miRNAs, previously described as upregulated in the serum of hepatocellular carcinoma (HCC) patients [133], and their specific roles in the promotion of angiogenesis in HCC cells [134]. Using HCC cell lines such as QGY-7703, HepG2, SKHep-1, and Huh-7, they discovered that only 5 miRNAs are secreted in the medium of HCC cells. Among those 5, higher levels of miR-210 correlated with increased microvessel density in HCC patients. The treatment of HCC cell lines with the programmed cell death 6-interacting protein (ALIX) and/or HRS, key components of exosome secretion, decreased the levels of miR-210 in the HCC-conditioned medium, indicating its secretion into exosomes. Exosomes, isolated from both patients’ sera and HCC cell lines, were incubated with HUVECs. Both types of exosomes stimulated HUVECs to form capillary-like structures, but when HCC cell lines were treated with a DROSHA inhibitor, essential for miRNA maturation, exosomes were no longer capable of promoting tubulogenesis. The same inhibitory effect was obtained by treating either HUVECs or HCC cell lines with anti-miR-210. In addition, the injection of HCC exosomes into nude mice confirmed their angiogenic activity. Mothers against decapentaplegic homolog 4 (SMAD4) and STAT6, two angiogenesis-associated factors, were considered to be putative miR-210 target genes. In fact, the treatment with exosomes from HCC patients’ sera or HCC cell lines reduced the levels of these proteins, whereas the incubation with exosomes from anti-miR-210 transfectants did not change SMAD4 and STAT6 levels, reducing the tube formation capability in HUVECs instead. These results highlight the role of miR-210, contained in HCC exosomes, in the angiogenic stimulation of endothelial cells.

EVs derived from non-CSC tumor cells contribute to create an immunosuppressive TME [135]. In particular, a dysfunction of tumor-resident CD8^+^ T cells was reported. This dysfunction, partially attributed to tumor-derived exosomes (TDEs), is characterized by the loss of CD27 and CD28, programmed death receptor 1 (PD-1), and T cell immunoglobulin and mucin protein 3 (Tim-3) [136]. Maybruck et al. [137] observed that tumor cells release a soluble factor able to induce a suppressor phenotype (SP) in human CD8^+^ T cells [135,138,139]. They consequently hypothesized that TDEs were the soluble factors contributing to the SP of CD8^+^ T cells. In particular, isolated CD8^+^ T cells from patients affected by head and neck cancer showed a low CD27/CD28 expression, indicating an SP. The neighboring T cell function was inhibited by the incubation of these cells with blood T lymphocytes, purified from the same patients of the responder cells. Head and neck carcinoma tumor cell lines such as Tu167, SCC0209, and HN60 secreted exosomes that induced SP in CD8^+^ T cells. However, human colorectal adenocarcinoma cell line Caco2 did not initiate this process, suggesting that not all cell lines are able to induce T cell dysfunction. After demonstrating the incorporation of TDEs into CD8^+^ T cells, they attributed T cell dysfunction to galectin-1 (Gal-1), an exosomal protein that is involved in this immunoregulation. Galectin-1 knockdown reduced exosome capacity to induce a T cell suppressive phenotype. Moreover, they showed that purified RNA from Tu167 exosomes is able to induce SP when transfected into normal donor CD8^+^ cells [137]. Those data documented the important role of TDEs in immune dysfunctions, suggesting them as possible therapeutic targets to prevent T cell dysfunction and exert an effective antitumor immune response.

On the other hand, EVs secreted by tumor stroma can influence tumor progression by acting on non-CSC tumor cells [50]. To identify which factors are delivered from CAF exosomes into colon rectal cancer (CRC) cells, Hu et al. isolated CAFs and normal fibroblasts (NFs) from colorectal cancer tissues and normal colorectal mucosa, respectively [140]. The growth medium (GM) of both cell types was collected and used to treat human CRC cell lines SW480, SW620, and LOVO, finding that cells treated with CAFs-CM had an increased migration and invasion ability, as well as chemotherapy resistance. Based on previous results, which indicated exosomes as important mediators in tumor metastasis and chemotherapy resistance [141], they isolated exosomes from CAFs and NFs and set up an in vitro experiment to evaluate their effects on CRC cell lines. Results showed that both CAF-CM and CAF exosomes increased the migration and invasion of CRC cells. The same result was obtained in vivo: mice injected with CRC cells, which had been treated with CAF-CM and CAF exosomes, formed more and larger lung metastasis nodules. In addition, they observed that CAF exosomes mainly contained miR-92a-2p and that, when incubated with CRC cells, miR-92a-3p expression was increased. They identified F-box and WD repeat domain-containing 7 (FBXW7) and modulator of apoptosis 1 (MOAP1) as the downstream targets of miR-92a-3p. In fact, FBXW7 and MOAP1 significantly decreased in CRC cells treated with CAF exosomes. The introduction of miR-92a-3p in CRC cells reduced FBXW7 and MOAP1 levels, incrementing β-catenin and lessening mitochondrial apoptosis. To determine the clinical role of miR-92a-3p, its expression was analyzed in normal and cancer tissues from CRC patients, showing that the expression was increased in patients with CRC metastasis and fluorouracil/oxaliplatin (5-FU/L-OHP) therapy resistance and inversely correlated with FBXW7 and MOAP1 [140]. Those findings suggest how CAFs can induce tumor progression and metastasis by releasing miR-92a-3p-enriched exosomes into the TME, and also underline that miR-92a-3p inhibition may be used as a potential therapeutic strategy to prevent CRC metastasis.

Different studies indicated that CAFs also play an important role in cancer initiation, angiogenesis, invasion, and metastasis of breast cancer [142,143]. Recently, some studies reported that miRNAs contained in the exosomes are incorporated into recipient cells, stimulating oncogenic signaling [144,145]. In this light, Donnarumma et al. investigated the role of specific miRNAs contained in CAF exosomes in the promotion of an aggressive phenotype in breast cancer cells [146]. Human breast biopsies were collected to obtain NFs and CAFs. Exosomes have been isolated from both cell populations and used for a genomewide analysis to identify miRNAs differently expressed between the two samples, and an upregulation of miR-21, miR-378e, and miR-143 was detected in CAFs. To determine whether exosomes could transfer these miRNAs to breast tumor cells, NF and CAF exosomes were coincubated with a breast cancer cell line, T47D, demonstrating that they were taken up by T47D cells. Interestingly, the expression of miR-21, miR-378e, and miR-143 was upregulated in T47D cells when coincubated with CAF exosomes. Moreover, they exhibited an increased ability to form mammospheres, anchorage-independent cell growth, and EMT markers, but these effects were reversed when T47D cells were transfected with specific anti-miRNAs. To confirm whether CAF exosomes induced stemness and EMT phenotype through specific miRNAs, they transfected NFs with miR-21, miR-378e, and miR-143 and isolated their exosomes to treat T47D cells, thus obtaining the same effects as CAF exosomes [146].

**Figure 1:**
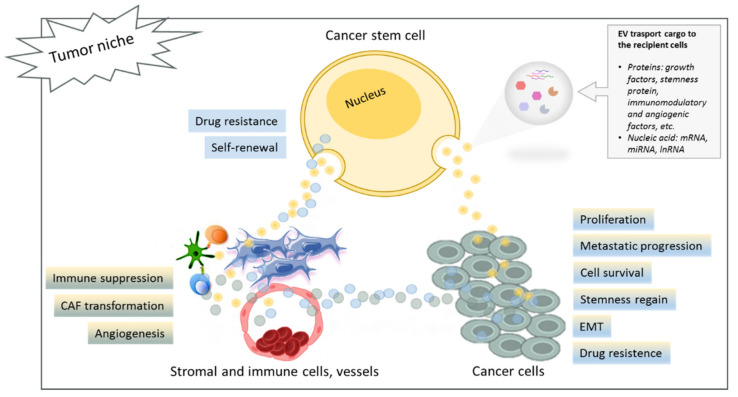
Schematic representation of the communication between CSCs and tumor niche showing activated mechanisms induced by the transfer of EV content to the recipient cells. Abbreviations: CAF, cancer-associated fibroblast; EMT, epithelial–mesenchymal transition.

MiR-409 was identified by Josson et al. as another important miRNA secreted by CAF exosomes in prostate cancer (PCa). MiR-409 was able to induce EMT and stemness regain in epithelial cancer cells [147]. They carried out a miRNA profiling on normal and cancer fibroblasts isolated from prostatic cancer patients. Results showed that the most upregulated miRNA in CAFs was miR-409-3p, which localized in a specific cluster called DLK1-DIO3, known to be associated with embryogenesis, pluripotent stem cell formation, and tumorigenesis [148]. Correlating miR-409-3p expression with the tumor grade, they discovered that stromal miRNA higher expression was correlated to a higher tumor grade. To better understand the function of miR-409-3p in CAFs and PCa progression, they overexpressed miR-409-3p in CAFs from PCa patients, obtaining an increased expression of EMT and metastasis markers. The release of those miRNAs from CAFs occurred through EV transfer, and when PCa cell lines were incubated with stromal EVs, an internalization of those vesicles, as well as miR-409-3p, was observed. There was a consequent decrease in miRNA target genes and an increase in EMT markers and proliferation. Increased tumor growth and EMT progression were also confirmed in vivo, inoculating PCa cells and stromal fibroblasts (that expressed miR-409-3p) into athymic mice [147]. Altogether, those findings highlight the role of CAF exosomes and their specific miRNAs in stemness regain, EMT, and tumor progression. However, further investigation is needed to confirm these promising results and eventually develop new anticancer therapies based on EV targeting. A representative scheme of the CSC cross-talk on tumor bulk is reported in Figure 1.

## 5. EV-Based Strategies for CSC Targeting as Anticancer Therapy

We have already discussed the role of CSCs in tumor development and progression and their crucial activities in the homeostasis of TME, as well as in chemoresistance and immunotolerance. It has been reported that these processes are regulated by paracrine factors delivered by EVs to recipient cells, orchestrating the cell–cell communication within TME [50].

**Figure 2:**
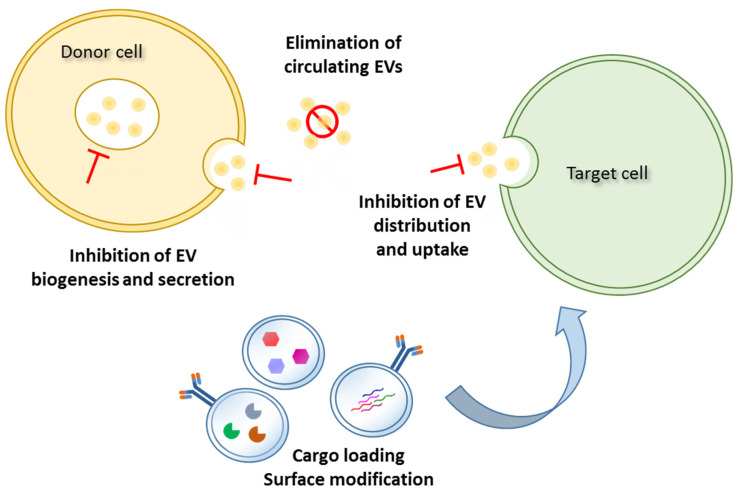
Schematic representation of EV-based strategies for CSC targeting and anticancer therapies.

Therefore, there is a considerable interest in the development of new anticancer therapies solely focused on EV-based strategies, such as the inhibition of biogenesis, the release or uptake of EVs, the removal of circulating cancer EVs, and the use of natural bioengineered or synthetic functionalized EVs to target CSCs and release anticancer drugs (Figure 2).

### 5.1. Inhibition of Biogenesis and Release/Uptake of EVs

Different studies have tested those strategies to prevent CSCs from transmitting chemoresistance to non-CSC tumor cells. Among them, the MEK1/2 inhibitor U0126 was proven effective in sensitizing the chemoresistant human pancreatic adenocarcinoma cells Suit-2 to the chemotherapeutic drug gemcitabine [149] by preventing MV secretion [150]. Similarly, GW4869, a neutral sphingomyelinase 2 (nSMase2) inhibitor, reduced exosome secretion, reversing chemoresistance in colorectal, pancreatic, and ovarian cancer cells [151,152,153]. Indomethacin, an anti-inflammatory drug inhibiting the lipid transport [154], was effective in the chemosensitization of lymphoma cells in vitro [155]. Ketotifen, another exosome release inhibitor, was able to sensitize the cervical cancer cells HeLa as well as MCF-7 and BT549 breast cancer cells to doxorubicin [156]. The chemotherapeutic fludarabine was found to inhibit the EV-driven cross-talk between breast CSCs and the local microenvironment [157]. Based on the lncRNA X-inactive specific transcript (lncRNA Xist) downregulation in brain metastases, Xing et al. silenced Xist in breast cancer cells, thus promoting brain metastatic growth in xenograft models through the stimulation of the epithelial–mesenchymal transition and the activation of the mesenchymal–epithelial transition factor (c-Met), as well as the stemness regain of breast cancer cells. Breast cancer cells expressing Xist at low levels secreted miR-502-enriched EVs, which, through the M1–M2 polarization of microglia, inhibited T cell proliferation by upregulating the immunosuppressive cytokines. Fludarabine was demonstrated to be mainly selective for low-Xist-expressing breast cancer cells. The death of these cells resulted in the interruption of miR-502-enriched exosome release with the consequent inhibition of microglial M2 conversion, which suppresses T cell activities.

Another research group investigated how the interplay between oral squamous cell carcinoma CSC-derived EVs and the tumor microenvironment could determine cisplatin resistance [158]. In particular, they found that CSC-derived EVs (from oral cancer cell tumorspheres) are enriched with miR-21-5p, an important mediator in cisplatin resistance, [159,160] and a direct regulator of tumor suppressor genes, such as programmed cell death 4 (PDCD4) and PTEN. PDCD4 and PTEN, in turn, regulate pathways such as β-catenin, PI3K, STAT3, mTOR, and TGF-β1 [161]. In fact, CSC-derived EVs also contained high levels of β-catenin, PI3K, STAT3, mTOR, and TGF-β, all oncogenic molecules that are involved in metastatic progression, stemness regain, and poor survival in cancer patients [162,163]. The cocultures of CSC-derived EVs with parental oral cancer cells enhanced cell viability, stemness, clonogenicity, tumorsphere formation, and cisplatin resistance. In addition, the treatment with CSC-derived EVs was also able to induce the transformation of normal gingival fibroblasts into CAFs, favoring the oral cancer cell oncogenicity. However, the treatment with ovatodiolide (OV), a component of *Anisomeles indica*, was effective in counteracting the CSC-derived EV tumorigenic effects on oral cancer cells in terms of reduced viability, clonogenicity, stemness, tumorsphere formation, and cisplatin resistance. It also hindered CAF transformation by reducing the content of PI3K, STAT3, mTOR, TGF-β1, and miR-21-5p in CSC-derived EVs. In vivo, the treatment with OV, alone or in combination with cisplatin, was able to reduce the oncogenicity of tumorspheres, also decreasing the content of PI3K, STAT3, mTOR, TGF-β1, and miR-21-5p in CSC-EVs of tumor samples explanted from OV-treated mice.

The treatment with OV was also proven to be efficacious in suppressing the effects induced by exosome-derived colon cancer tumorspheres in vitro [164]. It was demonstrated that exosomes secreted by colon cancer tumorspheres induced 5-FU resistance, migration, and tumorsphere formation in the parental colon cancer HCT116 and HT29 cells. In addition, exosomes were able to stimulate the transformation of CAFs and the polarization of M2 macrophages in vitro. The content analysis of exosomes revealed that they were enriched with the well-known oncogenic molecules IL-6, STAT3, TGF-β1, β-catenin, and oncomiR-1246, which is one of the miRNAs upregulated in exosomes from colon cancer patients’ serum [165]. The treatment with OV was able to decrease exosomal cargos from tumorspheres, counteracting 5-FU resistance, migration, and tumorsphere formation of HCT116 and HT29 cells in cocultures. Moreover, exosomes from OV-treated tumorspheres were less efficient in inducing CAF transformation and M2 polarization, likely downregulating β-catenin activity. A xenograft model confirmed the ability of OV treatment to counteract tumor growth by inhibiting IL-6, STAT3, β-catenin, and exosomal miR-1246 in mice serum [164].

In a study by Chuang et al. [166], the analysis of the exosome content from glioblastoma multiforme (GBM)-associated macrophages (GAMs) revealed an enrichment with miR-21, which conferred a stemness phenotype and temozolomide (TMZ) resistance to GBM cell cocultures. Furthermore, not only GMB cell cocultures secreted IL-6 and TGF-β1, which promoted the M2 polarization of GAMs, but also they increased the expression of PDCD4 and of stemness markers, such as sex-determining region y (SRY) and region y-box 2 (Sox2), STAT3, nestin, and miR-21-5p. The use of the STAT3 inhibitor pacritinib counteracted GBM stemness phenotype acquisition, tumorsphere formation, and TMZ resistance. In addition, pacritinib treatment inhibited the M2 polarization and the release of miR-21-enriched exosomes by GAMs.

In a study conducted by Gernapudi et al. [167], mouse preadipocytes (3T3L1) were treated with a natural antitumoral agent, namely, shikonin (SK), and their exosomes were used for breast cell line co-cultures (MCF10DCIS). They found high levels of miR-140 in the 3T3L1-derived exosomes that stimulated differentiation, stemness, and migration of breast cancer cells through miR-140/SOX2/SOX9 axis. In addition, 3T3L1-derived exosomes were able to promote tumorigenesis in vivo. However, the treatment of 3T3L1 with SK inhibited their protumoral effects by targeting the SOX9 pathway. Those data showed that the targeting of EVs could be a possible effective strategy in tumor progression. However, although those results are promising, further investigations are needed to better clarify the specific biomolecular mechanisms underlying those inhibitory effects on CSC-EV biogenesis and release.

### 5.2. Removal of Circulating Cancer EVs

Hemofiltration was proposed as a possible strategy to remove tumor-derived EVs from peripheral blood [168]. In a mouse model of breast cancer, metastatic progression was reduced, thanks to the removal of EVs through antibodies against EV surface markers, such as CD9 and CD63 [169]. These findings support the strategy of inhibiting cancer metastasis by blocking cancer-derived EVs using antibodies against their surface markers. However, this procedure is not cancer specific, and EVs from normal body cells would also be targeted, preventing communication between healthy cells with adverse side effects. A novel technology, based on aptamer-functionalized nanoparticles to eliminate blood oncogenic EVs into the small intestine and to counteract oncogenic EV-induced lung metastasis in a mouse model, has been reported [170]. This technology utilized positively charged mesoporous silica nanoparticles designed with EGFR-targeting aptamers, which specifically bind the negatively charged EVs and drag them from the blood to the bile duct for elimination. In the wake of that, the removal of circulating HER2-expressing EVs from the plasma of breast cancer patients was also proposed to be a possible solution for overcoming the chemoresistance to Herceptin^®^ [168]. In addition, a phase I clinical trial using Hemopurifier^®^ in conjunction with pembrolizumab (Keytruda) in patients with advanced head and neck cancer has been recently approved by the Food and Drug Administration (FDA) (NCT04453046). Although these procedures demonstrated to be effective in removing oncogenic EVs from the blood, further investigations are needed to find specific oncogenic EV markers for each tumor type.

### 5.3. Natural Bioengineered EVs for CSC Targeting and Drug Delivery

As reported above, EVs are promising potential drug delivery systems for cancer therapies. Natural EVs possess innate targeting abilities and low immunogenicity; EVs are able to specifically deliver drugs to the tumor site. They are stable in the circulation and can naturally cross over biological barriers (even the blood–brain barrier) [171]. EVs can escape phagocytosis and possess endogenous systems for cargo uptake, trafficking, and release [171]. By exploiting their main characteristic to transmit nucleic acids and proteins to the recipient cells, it would be possible to interfere with or to neutralize some crucial CSC capacities.

It has been demonstrated that exosomes derived from osteogenic differentiated human adipose-derived stem cells (ASCs) are able to induce the osteogenesis of CSCs derived from CD133^+^ MG63 osteosarcoma cells [172]. The exosome treatment of CSCs upregulated the expression of osteogenic-related genes, and it was also effective in downregulating important drug-resistance genes, such as the ATP-binding cassette (ABC) transporter, the breast cancer gene family (BRCA1 and BRCA2), and the epidermal growth factor (ErbB) gene family. Naseri et al. [173] reported the use of mesenchymal-stem-cell-derived exosomes (MSCs-Exo) to deliver locked nucleic acid (LNA)-antimiR-142-3p into MCF7-derived cancer-stem-like cells. Indeed, LNA-antimiR-142-3p targets miR-142-3p and miR-150, which are associated with clonogenic and tumorigenic capabilities in breast cancer stem cells (BCSCs) [174]. They observed that MSCs-Exo could efficiently transfer LNA-antimiR-142-3p to BCSCs, reducing the expression of miR-142-3p and miR-150 as well as clone-formation and tumor-initiating abilities [173]. Those results support the use of MSC-derived exosomes as promising carriers to deliver anticancer molecules. PTX is a chemotherapeutic agent that acts as a microtubule-stabilizing drug, inhibiting cancer cell mitotic activity [175]. The release of PTX in the conditioned medium by loaded ASCs was effective in reducing CG5 breast cancer cell survival, proliferation, and clonogenicity in vitro. These findings were confirmed by the tumorigenesis inhibition in a mouse model. Those results strongly encourage the development of a new potential therapeutic strategy against cancer based on CSC targeting, reprogramming, and drug delivery.

However, different studies have raised some concerns about the use of stromal/mesenchymal cells or their derivatives as therapeutic agents in cancer patients [176,177]. That is why further experiments in vitro and in vivo are required to collect more evidence confirming the efficacy and safety of those strategies in different types of cancer.

### 5.4. Synthetic Nanoparticles for CSC Targeting and Drug Delivery

The use of synthetic vesicles is an emerging field in cancer research. Although, as previously described, natural extracellular vesicles have several advantages, artificial vesicles can be designed “ad hoc” to target specific CSCs and release a well-known cargo in a more controlled manner [13]. Apart from the desired enriched molecule, the entire content of natural EVs remains unidentified most of the time, and the effects of these unknown factors are impossible to predict. Liposomes, nanovesicles, polymeric micelles, and nanoparticles can home into the TME via the bloodstream, and thanks to their porous structure, they can specifically deliver anticancer drugs with low toxicity for nontargeted cells [13]. Moreover, cargos can bypass the gastrointestinal tract and liver with no alterations (i.e., degradation/metabolism), resulting in a reduced metabolic clearance and, at the same time, in an increased absorption and oral bioavailability [178]. The surface of synthetic vesicles can be modified “ad hoc” to reach and specifically target CSCs or TME, optimizing their distribution, promoting cellular uptake, and reducing the damage of healthy tissues. It is possible to modify surface charge or add peptides and antibodies, as well as loading bioactive molecules and small noncoding RNAs (miRNAs, siRNAs, and lncRNAs) as cargos [178]. In this regard, the use of the RGD peptide (arginine, glycine, aspartic acid) has been described to selectively bind the αvβ3 integrin receptor that is specifically expressed in cancer vessels [179]. A study by Yong et al. [180] reported the application of biocompatible tumor-cell-exocytosed exosome-biomimetic porous silicon nanoparticles (E-PSiNPs) as a drug carrier for chemotherapy, in particular doxorubicin (DOXO). DOX@E-PSiNPs can be obtained by exocytosis of the endocytosed DOXO-loaded PSiNPs from different tumor cell lines. They demonstrated that CSC spheroids (from mouse hepatocellular carcinoma cell line H22) were able to uptake DOX@E-PSiNPs, inhibiting tumor growth. Once intravenously injected in mice bearing H22 hepatocarcinoma (subcutaneous, orthotopic, and metastatic tumors), DOX@E-PSiNPs extravasate from the blood vessels into the tumor parenchyma, reducing tumor growth [180]. In another study by Sun et al., the authors demonstrated that doxorubicin-tethered gold nanoparticles were effective in delivering doxorubicin to breast CSCs. The treatment was able to reduce mammosphere formation and cancer initiation activity in vitro and inhibit tumor growth in murine models as well [181]. In a study by Ni et al. [182], the authors reported the effective use of CD133-tethered poly(lactic-co-glycolic acid (PLGA) nanoparticles loaded with salinomycin to target CD133+ osteosarcoma CSCs. CSCs were also killed by hyaluronic acid (HA)-coated PLGA nanoparticles carrying salinomycin (SLM) and paclitaxel (PTX) [183]. PLGA nanoparticles grafted with anti-CD133 monoclonal antibody were proven to counteract drug resistance and relapse of breast CSCs [184]. In another study, paclitaxel–hyaluronan bioconjugate (ONCOFIDTM-P, 20% drug content) was used in xenograft models to inhibit the tumorigenesis of human ovarian cancer IGROV-1 and OVCAR-3 overexpressing CD44 [185]. However, after systemic administration, HA-based drug delivery nanoparticles gathered in the liver. The conjugation of HA with poly PEG (ethylene glycol), on the other hand, seems to overcome this issue, while also prolonging the circulation time in the bloodstream [186]. Moreover, bortezomib-loaded PEG-b-PLA nanoparticles accumulate into the breast cancer bulk and target CSCs, inducing cell apoptosis and death [187]. Some works described the results obtained with a combination of drugs. For instance, Liu et al. [188] designed particular poly (ethylene glycol)-peptide-poly(*ε*-caprolactone) nanoparticles that delivered both miR-200c and docetaxel (DOC) to CSCs and non-CSC tumor cells, thus reducing tumor growth in the xenograft model.

Yang et al. [189] inhibited liver metastasis in a nude mouse model using docetaxel-encapsulated polylactic acid (PLA) nanoparticles previously conjugated to the high binding peptide CVKTPAQSC, which specifically targets lung cancer-stem-like cells (CSLCs). Bioreducible poly(beta-amino ester) nanoparticles were designed to embed miRNAs (nano-miRs) and treat glioblastoma (GMB). In particular, the intratumoral infusion of these nanomiRs, containing miR-148a and miR-296-5p, inhibited stem cell phenotype in vitro and reduced tumor growth in a xenograft model. In addition, the treatment was able to increase long-term survival from GBM and enhance the response to standard-of-care γ-radiations [190]. The use of nanoparticles embedded with siRNAs targeting oncogene polo-like kinase 1 (Plk1) in combination with LY364947, an inhibitor of the TGF-β type I receptor, strongly inhibited breast CSC proliferation in vitro and tumor growth in vivo [191] (see Table 1).

Although the study on these strategies is still in its early stages, some clinical trials based on nanoparticles as an anticancer drug delivery system have been approved, including albumin-bound paclitaxel particles (Abraxane), PEGylated liposome (Doxil), iron oxide nanoparticles (NanoTherm), PEG-1 asparaginase (Oncaspar), and methoxy-PEG-poly (d,l-lactide)-paclitaxel micelle (Genexol-PM) [13].

## 6. Conclusions and Perspectives

In recent years, the critical role of CSCs in tumor initiation, development, and metastasis was extensively investigated. The ability of EVs to orchestrate the tumor microenvironment and confer chemo- and radioresistance lies in the release of paracrine factors. Thus, different CSC-targeting strategies have been developed to treat malignancies by using either EV-inhibiting drugs or EVs loaded with specific anti-CSC bioactive molecules. EVs display intrinsic targeting and drug delivering abilities with less immunogenicity. However, the entire content of natural EVs remains unidentified most of the time, and the effects of these unknown factors are impossible to predict. Therefore, it is mandatory to clarify the specific biological functions of each natural EV component and further investigate the fundamental processes of EV biology, cargo loading, and specific cell targeting. In addition, the efficacy and specificity of some inhibitors can vary between different cell lines and cell types, consequently pointing to the use of a combination of inhibitory molecules as a possible solution. One of the main issues is the possible adverse effects (cytotoxicity) of those treatments. For instance, EV inhibitors may be toxic for healthy tissues, disrupting the physiological cell–cell communication. Moreover, some of these EV strategies are not cancer specific, so EVs from normal body cells would also be targeted, preventing communication between healthy cells with adverse side effects.

In recent years, researchers focused their attention on synthetic nanoparticle-based therapies for CSC targeting. Artificial nanoparticles can be designed “ad hoc” to target specific CSCs and release a well-known cargo in a more controlled manner. Nanoparticles can also be rebuilt with specific surface antigens that selectively bind CSC markers. The possibility of modifying the chemical or physical characteristics of synthetic EVs in order to optimize the loading of different drugs and selectively target specific CSC markers represents an evident advantage in terms of targeting efficacy and tumor bulk penetration. Although some clinical trials based on nanoparticles as an anticancer drug delivery system have been approved, many issues remain to be addressed. Passage through the gastrointestinal tract and liver (i.e., degradation/metabolism) and metabolic clearance could interfere with the absorption and oral bioavailability. EV distribution, cellular uptake, and prevention of healthy tissue damage represent the main issues to deal with. These innovative strategies need the use of appropriate in vitro and in vivo models in order to overcome those limitations and optimize CSC targeting for cancer therapy.

## Figures and Tables

**Table 1:** EV-based strategies for CSC targeting.

Strategy	Agent/Method	Tumor Type	Inhibition	References
EV biogenesis and release/uptake	U0126	Human pancreatic adenocarcinoma cells Suit-2	Gemcitabine resistance	[149]
	GW4869	Colorectal, pancreatic, and ovarian cancer cells	Chemoresistance	[151,152,153]
	Indomethacin	Lymphoma cells	Chemoresistance	[155]
	Ketotifen	HeLa, MCF-7, and BT549 breast cancer cells	Doxorubicin resistance	[156]
	Fludarabine	Metastatic breast cancer	M2 polarization	[157]
	Ovatodiolide	Oral squamous cell carcinoma	Cisplatin resistance	[158]
		Colon cancer HCT116 and HT29 cells	5-FU resistance, CAF transformation, and M2 polarization	[164]
	Pacritinib	Glioblastoma multiforme	Temozolomide resistance, M2 polarization	[166]
Removal of circulating EVs	Shikonin	Breast cancer MCF10DCIS cells	Differentiation, stemness, and migration of cancer cells	[167]
	Anti-EV marker antibodies	Mouse model of breast cancer	Metastasis	[170]
	Hemofiltration in conjunction with pembrolizumab	Advanced head and neck cancer patients (NCT04453046)	Metastasis	[168]
Use of natural bioengineered EVs	EVs from osteogenic differentiated hASCs	MG63 osteosarcoma cells	Osteogenic differentiation of CSCs	[172]
	EVs from mMSCs loaded with LNA-antimiR-142-3p	MCF7-derived cancer-stem-like cells	Mammosphere formation in vitro and tumorigenicity in vivo	[173]
	CM from PTX-primed hASCs	CG5 breast cancer cells	Proliferation in vitro and tumor growth in vivo	[175]
Use of synthetic NPs	DOX@E-PSiNPs	Mouse hepatocellular carcinoma cell line H22	Tumor growth	[180]
	DOXO-tethered gold nanoparticles	Mouse breast cancer cells	Sphere formation	[181]
	CD133-tethered PLGA nanoparticles—salinomycin loaded	CD133^+^ osteosarcoma and breast cancer	Proliferation, drug resistance, and relapse of breast CSCs	[182]
	PTX-hyaluronan bioconjugate (ONCOFID^TM^-P)	Human ovarian cancer IGROV-1 and OVCAR-3 overexpressing CD44	Tumor growth in vivo	[185]
	PEG-b-PLA nanoparticles loaded with bortezomib	Breast cancer cells	Proliferation and survival	[187]
	PEG-peptide-poly(e-caprolactone) nanoparticles loaded with DOXO and miR-200c	Gastric carcinoma cell line BGC-823	Tumor growth in vivo	[188]
	PLA nanoparticles—peptide CVKTPAQSC—docetaxel loaded	Lung-cancer-stem-like cells	Liver metastasis in a mouse model	[189]
	Poly (beta-amino ester) nanoparticles loaded with miR-148a/miR-296-5p	Glioblastoma cells	Stemness in vitro, tumor growth, and sensitizing to γ-radiation in vivo	[190]
	Nanoparticles with siRNAs targeting Plk1 in combination with LY364947	Breast CSCs	Proliferation in vitro and tumor growth in vivo	[191]

Abbreviations: 5-FU, 5-fluorouracil; CAF, cancer-associated fibroblasts; CM, conditioned medium; NPs, nanoparticles; hASCs, human adipose-derived stem cells; mMSCs, mouse mesenchymal stem cells; PTX, paclitaxel; DOX@E-PSiNPs, endocytosed doxorubicin-loaded exosome-biomimetic porous silicon nanoparticles; DOXO, doxorubicin; PLGA, poly(lactic-co-glycolic acid; PEG, polyethylene glycol; PLA, polylactic acid.
